# A finite element analysis of the carpal arch with various locations of carpal tunnel release

**DOI:** 10.3389/fsurg.2023.1134129

**Published:** 2023-05-03

**Authors:** Lu Yu, Jingyi Jia, Kishor Lakshminarayanan, Yiming Li, Yaokai Gan, Yifei Yao

**Affiliations:** ^1^ School of Biomedical Engineering, Shanghai Jiao Tong University, Shanghai, China; ^2^ Department of Sensors and Biomedical Engineering, Vellore Institute of Technology, Vellore, Tamil Nadu, India; ^3^ Orthopedic Department, Shanghai Ninth People’s Hospital, Shanghai Jiao Tong University School of Medicine, Shanghai, China

**Keywords:** carpal tunnel release, carpal arch compliance, transverse carpal ligament, finite element analysis, median nerve compression

## Abstract

**Objective:**

The purpose of this study was to investigate the effects of the location of transverse carpal ligament (TCL) transection on the biomechanical property of the carpal arch structure. It was hypothesized that carpal tunnel release would lead to an increase of the carpal arch compliance (CAC) in a location-dependent manner.

**Methods:**

A pseudo-3D finite element model of the volar carpal arch at the distal carpal tunnel was used to simulate arch area change under different intratunnel pressures (0–72 mmHg) after TCL transection at different locations along the transverse direction of the TCL.

**Results:**

The CAC of the intact carpal arch was 0.092 mm^2^/mmHg, and the simulated transections ranging from 8 mm ulnarly to 8 mm radially from the center point of the TCL led to increased CACs that were 2.6–3.7 times of that of the intact carpal arch. The CACs after radial transections were greater than those ulnarly transected carpal arches.

**Conclusion:**

The TCL transection in the radial region was biomechanically favorable in reducing carpal tunnel constraint for median nerve decompression.

## 1. Introduction

Carpal tunnel syndrome (CTS) is currently the most common upper-extremity compression neuropathy with prevalence rates varying between 1% and 5% of the general population in the United States (1). Elevated carpal tunnel pressure is commonly noted as the cause for the median nerve neuropathy (2–4). Structurally, the carpal tunnel is formed by the transverse carpal ligament (TCL) as its volar border and the interconnected carpal bones as its medial, lateral, and dorsal borders (5). The tunnel is crowded with the median nerve and nine digital flexor tendons, predisposing the median nerve to mechanical compression by the TCL (6). Clinically, if a conservative treatment fails, a surgical release of the carpal tunnel through TCL transection is performed so that the carpal tunnel becomes more compliant to accommodate the elevated pressure.

As a standard surgical treatment for carpal tunnel syndrome, carpal tunnel release (CTR) increases carpal tunnel volume (7–9), thereby decreases carpal tunnel pressure (10) and restores median nerve shape (11). The surgical approaches are mainly divided into two aspects: the open carpal tunnel release (OCTR) and endoscopic carpal tunnel release (ECTR). Surgical release of the transverse carpal ligament for the treatment of posttraumatic median nerve compression was first described in 1933 (12). The split of the transverse carpal ligament in a patient with CTS using endoscopy for the first time was not reported until 1987, and the endoscopic surgical approach has since been introduced (13).

Omokawa et al. investigated the anatomical course of the ulnar artery and its branches in relation to the TCL, as well as the position of the median nerve in 24 fresh cadaver hands. Their findings suggested that when performing surgery, transecting the TCL at a point roughly 5 mm radial to the radial margin of the hook of hamate may reduce postoperative bleeding and prevent inadvertent damage to the blood vessels and nerves (14). Z-lengthening is a surgical technique that has been developed as an alternative to the traditional complete incision carpal tunnel release surgery (15). In the Z-lengthening procedure, the surgeon creates a series of cuts in a zigzag pattern on the transverse carpal ligament to increase the available space for the compressed median nerve. Unlike complete severing, the Z-lengthening technique ensures that the ligament remains intact. Compared to the traditional carpal tunnel surgery, the Z-lengthening technique offers several advantages such as a smaller incision resulting in reduced pain and faster healing. In addition, this procedure has a lower incidence of complications such as damage to nearby nerves or blood vessels. As a result, Z-lengthening is an effective and safe surgical approach to treating carpal tunnel syndrome that can significantly enhance the quality of life for those affected by this condition (16). A previous study proposed a modified Z-lengthening technique that includes a distal flap on the radial side and a proximal flap on the ulnar side to avoid the hamulus insertion in the hamate bone and improve outcomes for carpal tunnel syndrome (17, 18). A meta-analysis of randomized controlled trials supported the effectiveness of Z-lengthening over the conventional TCL release for long-term functional improvement (19). Some research studies show better manual function and short-term grip strength with preserved TCL continuity comparing to complete cut of the ligament (20, 21). Therefore, TCL Z-lengthening has been proposed to preserve the TCL continuity and the first flexor tendon pulley, which is more effective than complete division. The ECTR has smaller cut incisions and better cosmetic results than the OCTR, but also has higher technical barriers and is associated with incomplete release of the transverse carpal ligament and neurovascular injury (16).

Notably, the volar carpal arch formed by the TCL contributes to the majority (93%) of the postoperative increase in the tunnel area (8). In a cadaveric study of the relationship between carpal tunnel cross-sectional area and intratunnel pressure, Kim et al. showed that carpal tunnel compliance after carpal tunnel release was nine times of that in the intact carpal tunnel (22).

Currently, the finite element (FE) model of the carpal tunnel is mainly pseudo-3D. Liong et al. established a patient-specific finite element model to analyze the relationship between repetitive finger flexion and the stress experienced on the nerve. The results show that the tendon volar movements (index finger and thumb flexion) impose larger stresses on the nerve than dorsal movements (middle finger flexion) (23). Walia et al. established a planar geometric model of carpal bones at the hamate level to analyze the best direction of the force for the maximization of the carpal arch area (CAA), so as to decrease the median nerve compression. The results showed that the maximal area occurred at 138° (volar-radial) relative to the hamate-to-trapezium axis (24). Mouzakis et al. simulated the computer work effects on the carpal arch area through finite element analysis. The results showed that the mouse work can introduce large deformation in the median nerve area, and the keyboard work can introduce a considerable and uneven axial lengthening of the nerve (25). Although there are many finite element models for studying carpal tunnel syndrome, they are currently mainly used to simulate the onset of symptoms, and few of them simulate the procedure of release surgery.

Although carpal tunnel release is a commonly performed surgical procedure for symptom relief in patients with CTS, the location of TCL transection can vary, and the implication of the transection variation on carpal tunnel biomechanics is unclear. The purpose of the current study was to investigate the effect of the location of TCL transection on the structural compliance of the distal carpal tunnel using a FE model. We hypothesized that carpal arch compliance (CAC) at the distal carpal tunnel would be dependent on the transection locations.

## 2. Materials and methods

### 2.1. Finite element modeling

**Figure 1:**
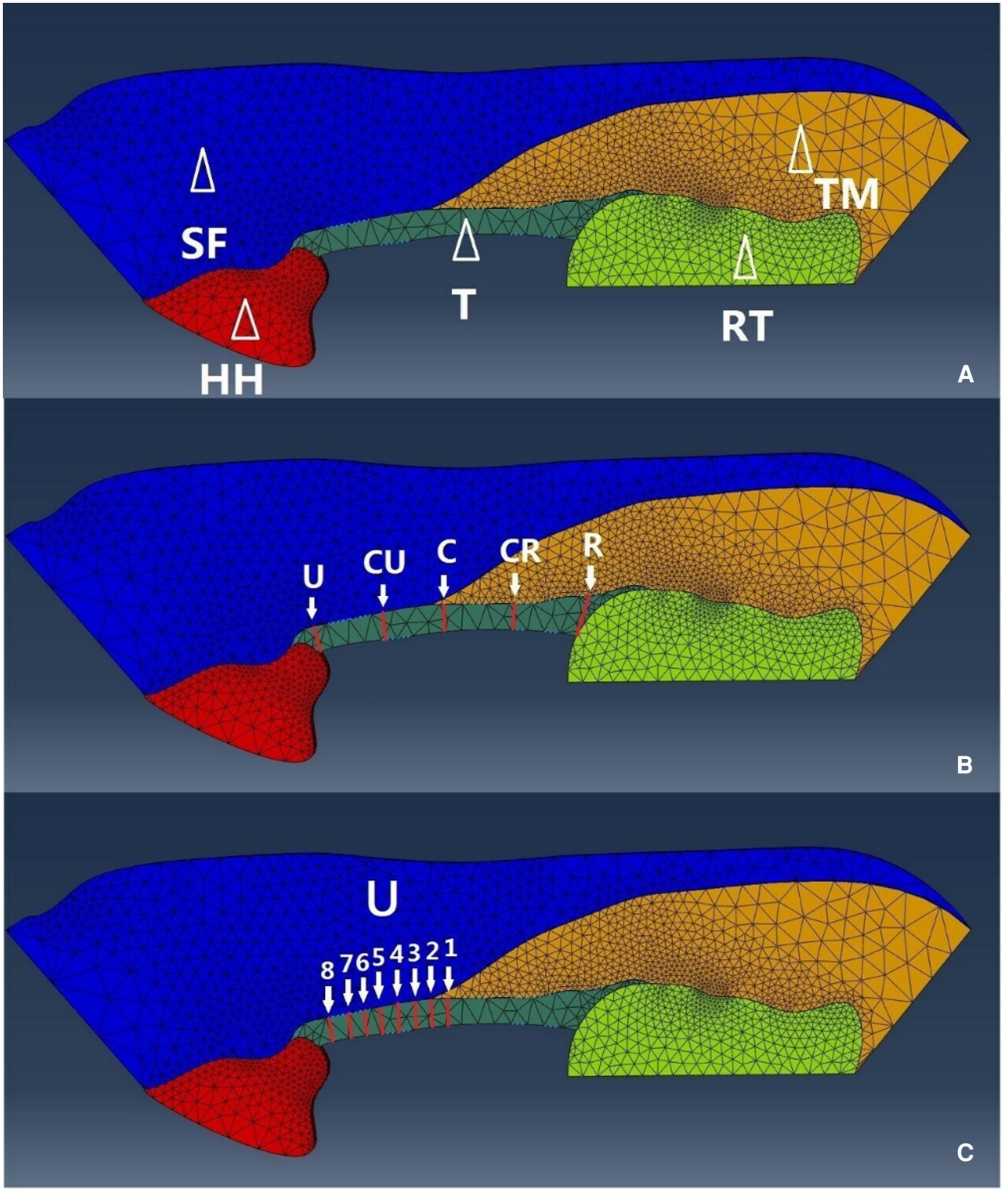
(**A**) The FE model of the volar carpal tunnel based on ultrasound image. The FE model components including the hamate bone, trapezium bone, thenar muscles, skin–fat, and TCL. SF, skin–fat; HH, hook of hamate; T, TCL; RT, ridge of trapezium; TM, thenar muscles. (**B**) Transection location on the TCL marked as U, ulnar; CU, central-ulnar; C, central; CR, central-radial; R, radial. (**C**) Transection location marked from 1 to 8 on the ulnar half of the TCL. FE, finite element; TCL, transverse carpal ligament.

The FE analysis in the current study was based on our previously developed pseudo-3D volar carpal arch structure at the distal tunnel level using computer-aided design modeling (SolidWorks 2012, Dassault Systems, Waltham, MA, United States) and then being exported to ABAQUS CAE (v6.10, Simulia, Providence, RI, United States) for FE analysis (26). Briefly, the model included the hamate bone, trapezium bone, thenar muscles, skin, fat, and TCL (Figure 1A). The skin and fat were modeled as a single skin–fat tissue. All tissue parts were manually segmented on transverse planar B-mode ultrasound images of the distal carpal tunnel of a cadaveric hand (male; left; age 74 years; height 177 cm; weight 95 kg). The specimen that had been thawed at room temperature prior to the test was placed in a supine, anatomically neutral position on the wrist. High-frequency (17 MHz) B-mode ultrasound images were acquired distal to the carpal tunnel using a linear array 18L6 HD transducer aligned in the transverse plane of the distal carpal tunnel along the line connecting the hook of hamate and ridge of trapezium. After acquiring the ultrasound images, the contours of thenar muscles, skin, fat, TCL, and the volar boundary of the hamate bone and trapezium bone were manually segmented and extracted with the use of the gray value threshold in ImageJ 1.46r (National Institutes of Health, Bethesda, MD, United States).

The elastic modulus of the hamate and trapezium bones was assumed as 10 GPa with a Poisson’s ratio of 0.3 (27). The TCL was modeled as linearly elastic and anisotropic with a volar-dorsal elastic modulus of 0.5 MPa and a transverse elastic modulus of 5.5 MPa (28) with a Poisson's ratio of 0.4. Hyperelasticity of both muscle and subcutaneous tissue was determined by a neo-Hookean strain energy potential equation with an effective Poisson's ratio of 0.49. The initial shear modulus of muscle was assumed to be 0.00425 MPa (29). The initial shear modulus of skin–fat was assumed to be 0.016 MPa (30). The intratunnel pressure was varied from 0 to 72 mmHg (0, 12, 24, 36, 48, 60, and 72 mmHg) in the simulation. The stiffness of the hamate-to-trapezium was set at 11.8 N/mm transversely and 2.9 N/mm in the volar-dorsal direction (31). The interface between any two components was assumed as a “tie” contact condition. The hamate, trapezium, TCL, muscle, and skin–fat tissue were all modeled as C3D10 quadratic tetrahedral elements. Displacement boundary conditions (free in-plane and fixed out-of-plane) were applied at both the distal and proximal surfaces of all components. Other surfaces were assumed free as their displacement boundary conditions. The hamate bone was fixed in all six degrees of freedom.

**Figure 2:**
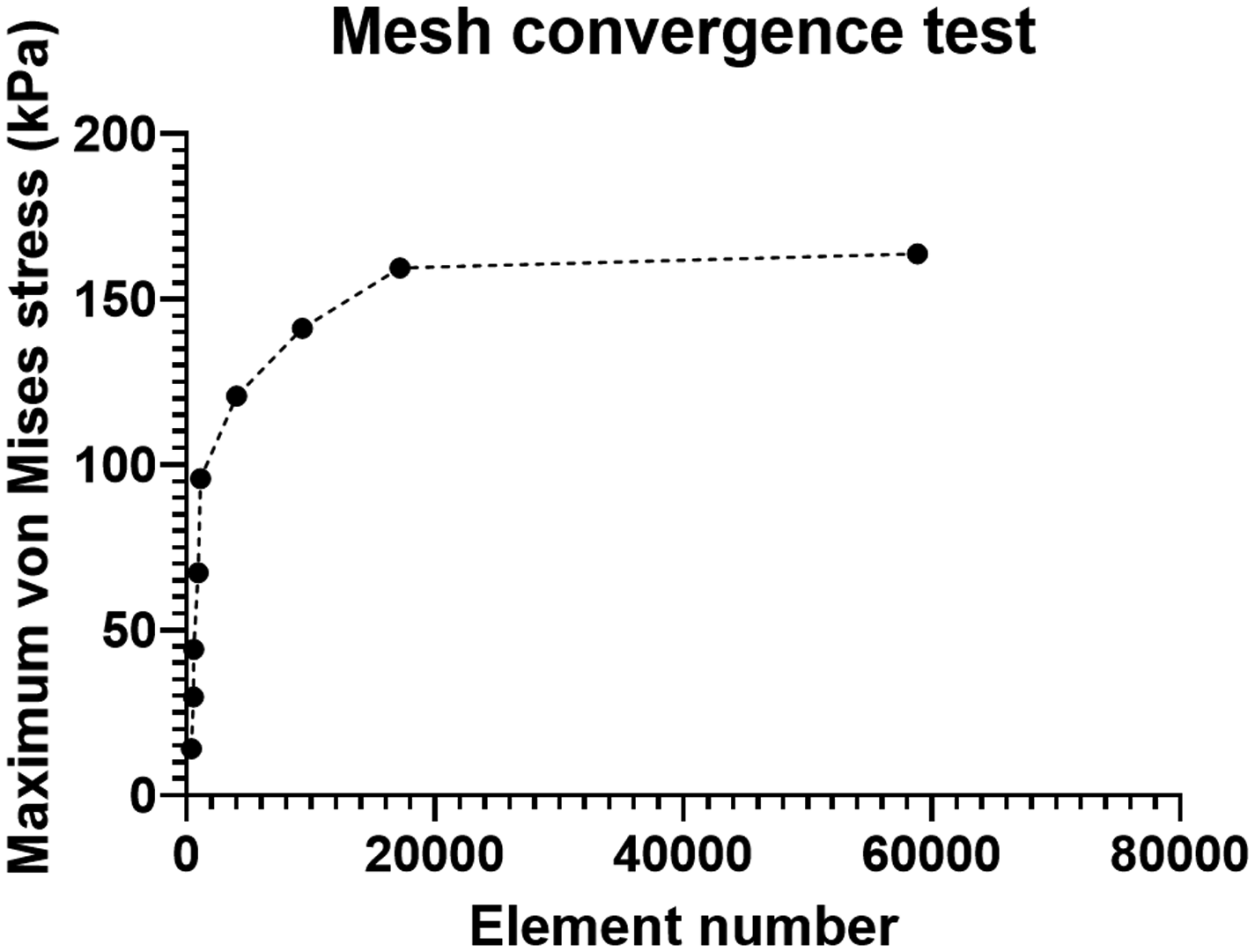
Mesh convergence test.

A mesh convergence test was conducted on the entire model, with the maximum von Mises stress on the TCL as the reference. The results indicated that when the global size was less than 0.3, the variation of von Mises stress was within 5% (Figure 2). Therefore, a global size of 0.3 was selected with 17,165 elements in the model.

### 2.2. Transection of TCL

We simulated two sets of TCL transection locations. For the first set, the transections were performed at the central location (C), and 4 and 8 mm radially (CR, R) and ulnarly (CU, U) from location C (Figure 1B). The second set of transections was performed on the ulnar part of the TCL with 1 mm increment from the central location, marked as U1–U8 (Figure 1C). A mesh convergence analysis was performed using h-refinement method, where around double and triple element numbers in all components in the model with the same boundary conditions and material properties showed within 2.6% and within 3.6% error, respectively, on the result of TCL tensile strain for each transection. The carpal arch areas of the intact and transected carpal tunnels under various intratunnel pressures were determined as the area formed by the dorsal TCL boundary and the line connecting the two points at the hook of hamate and ridge of trapezium. Linear regression analyses were performed on the carpal arch area as a function of intratunnel pressures for each TCL transection. The CAC was defined as the slope of the regressed linear equation.

### 2.3. Model validation with pressure regulation

The hand was positioned in a supinated and neutral orientation and dissected minimally, preserving the tunnel and volar soft tissues. An incision was made 4 cm distal to the distal wrist crease and directly proximal to the second web space, allowing for the insertion of a custom-designed medical air balloon (Advanced Polymers Inc., Salem, NH, United States) to apply artificial pressure within the tunnel. The balloon was aligned along the tunnel's longitudinal axis with the assistance of an ultrasound and extended beyond the proximal and distal edges of the TCL (32). The balloon was pressurized, and a pressure gauge was used to monitor the pressure, which was set at levels ranging from 0 to 300 mmHg. At each pressure level, a B-mode ultrasound image was taken at the transverse plane of the distal carpal tunnel, along the line connecting the ridge of the trapezium and the hook of hamate. The tensile strain of the TCL was analyzed by manual tracing using the multipoint selection tool in ImageJ. The predictions of the TCL tensile strain simulation were compared to the measurements obtained from the cadaveric specimen to validate within 5% difference compared to the finite element analysis approach.

## 3. Results

**Figure 3:**
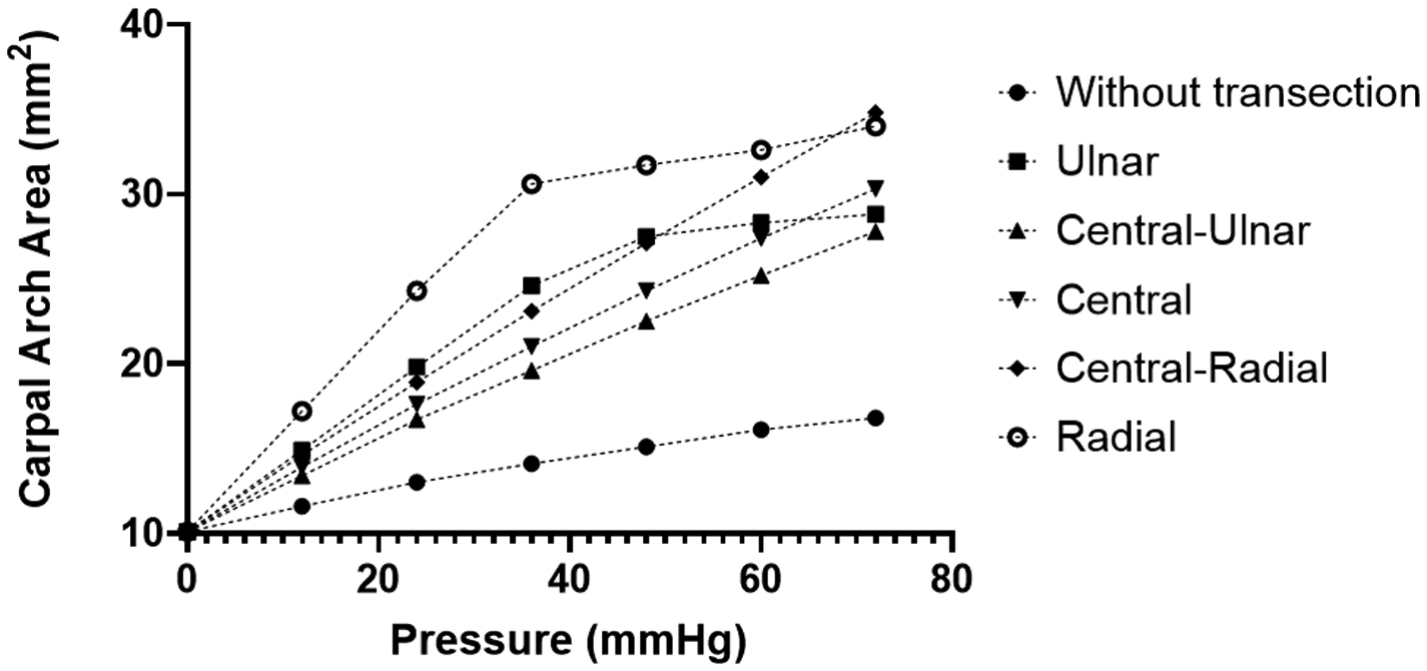
CAA with different intratunnel pressures for the intact tunnel and transected tunnel at both radial and ulnar sides. CAA, carpal arch area.

Analysis of the CAA under different intratunnel pressures at five locations (Ulnar, Central-Ulnar, Central, Central-Radial, and Radial) along the transverse direction on the distal TCL is shown in Figure 3. The linear regressions on the curve of the Central-Ulnar, Central, and Central-Radial TCL transections showed a CAC of 0.25, 0.28, and 0.34 mm^2^/mmHg, respectively, within the intratunnel pressure range of 0–72 mmHg (R-square value = 1.00 for each curve). The linear regressions on the curve of the Ulnar and Radial TCL transections showed 0.26 and 0.32 mm^2^/mmHg, respectively, as CAC within the intratunnel pressure range of 0–72 mmHg (R-square value = 0.90 and 0.84 for the Ulnar TCL transection and Radial TCL transection, respectively). CAC without TCL transection was 0.092 mm^2^/mmHg. Below 68 mmHg intratunnel pressure, radial TCL transection increased CAA the most among the five transection locations in Figure 3. Above 68 mmHg intratunnel pressure, Central-Radial TCL transection increased CAA the most (Figure 3). Central-Ulnar TCL transection increased CAA the least within the intratunnel pressure range of 0–72 mmHg. CAA increased by 18.03 mm^2^, a 107% increase, below 72 mmHg intratunnel pressure at the Central-Radial TCL location after transection. CAA only increased by 10.99 mm^2^, an increase of 65%, below 72 mmHg intratunnel pressure at the Central-Ulnar TCL location after transection.

**Figure 4:**
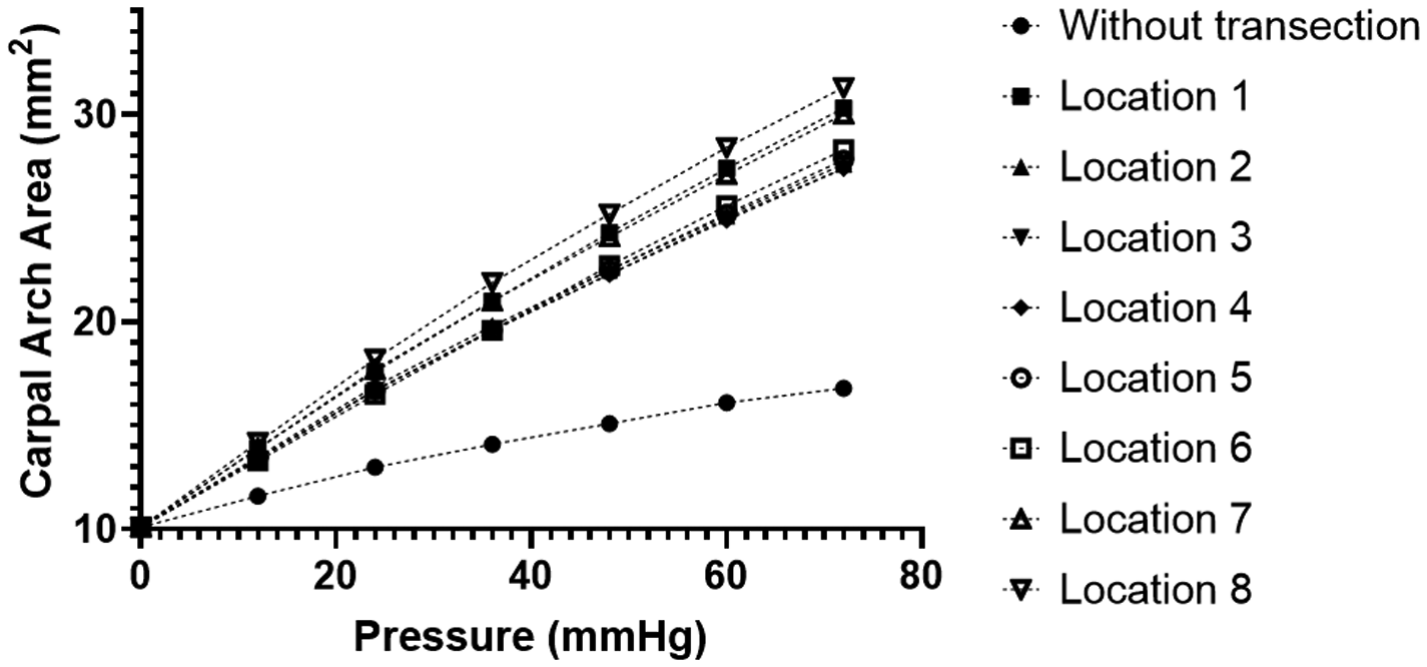
CAA with different intratunnel pressures for the intact and ulnarly transected tunnel conditions. CAA, carpal arch area.

During the traditional CTR, the TCL is transected on its ulnar side to avoid damage to the thenar muscle and median nerve. Analysis of CAA under different intratunnel pressures at the eight locations (Location 1–8) along the transverse direction on the ulnar half of the TCL is shown in Figure 4. The linear regressions on the curve of TCL transection at Locations 1–8 showed CAC of 0.28, 0.24, 0.24, 0.24, 0.25, 0.25, 0.27, and 0.29 mm^2^/mmHg, respectively, within the intratunnel pressure range of 0–72 mmHg (R-square value = 1.00 for each curve). There was no noticeable difference in CAC (within 0.05 mm^2^/mmHg) under TCL transection at Locations 1–8. At the intratunnel pressure of 72 mmHg, CAA increased by 28.77 mm^2^, an increase of about 70% compared to the condition without TCL transection.

## 4. Discussion

It is known that carpal tunnel release leads to an augmentation of the carpal tunnel space (7–9) and a reduction of the intratunnel pressure (3, 33). Our results of increased structural compliance after simulated TCL transections corroborate the biomechanical basis of the carpal tunnel release for median nerve decompression. A compliant carpal arch is more accommodating to elevated intratunnel pressure without excessive compression on the median nerve.

The current modeling results about CAC of the intact tunnel (0.092 mm^2^/mmHg) matched closely with a previous cadaveric study that examined the relationship between the intratunnel pressure and carpal arch area using MRI showing a compliance of 0.1 mm^2^/mmHg (32). The change in carpal tunnel compliance after carpal tunnel release was also previously studied by Kim et al. (22), showing an increase of 56.0 mm^2^ of the carpal arch area when the intratunnel pressure increased from 0 to 70 mmHg. In the current modeling study, the increase of the carpal arch area when pressure increased from 0 to 72 mmHg were 20.2 mm^2^ at the central location and 17.7–24.7 mm^2^ among all transection locations. Kato et al. reported that the carpal arch area increased by 82 mm^2^ (from preoperative 32 mm^2^ to postoperative 114 mm^2^) for patients who underwent carpal tunnel release. This discrepancy in the increase of the carpal arch area might be due to that the mechanical properties of the TCL surrounding tissues might not be the same as those in the cadaveric hands.

A finite element model is particularly advantageous for the study of the biomechanical effects of various transection locations. Carpal tunnel release is traditionally in the ulnar side of the TCL along the radial border of the ring finger, which likely corresponds to the simulated U1–U5 locations in the model. Our study showed that the CAA increase stayed relatively stable under transection at different locations on the ulnar half of the TCL. In this study, we found that the determination of TCL transection location on the ulnar half of the TCL in order to create the maximal carpal tunnel space is not a key factor for surgeon's consideration.

Along the transverse direction of the distal TCL, transection on the radial TCL increases CAA more than transection on the ulnar TCL. While in surgical practice, transection on the radial half of the TCL using OCTR or ECTR can lead to damage to the thenar muscles which attach on the radial TCL. Such damage to the thenar muscles and small nerve fibers might contribute to common CTR complications such as pillar pain and persistent pinch weakness. Future techniques with TCL transection at the edge of the trapezium might be a better approach to augment the carpal tunnel if the muscle and nerve fibers can be preserved intact, which might lead to better postoperative outcomes. Recently, the OCTR with Z-lengthening reconstruction of the TCL mobilizing the ulnar and radial flaps of the transverse carpal ligament with proximal release of the ulnar flap and distal release of the radial flap showed postoperative advantages (Seitz and Lallb 2013). Z-lengthening reconstruction of the TCL releasing the radial distal TCL might create more carpal tunnel space at the distal tunnel level. The effect of radial TCL transection on carpal tunnel morphology might be a promising direction for further studies. Our study aimed to investigate the distal portion of the carpal tunnel, with the goal of establishing a theoretical basis for determining the optimal location for the distal incision in future CTR surgeries, as well as for deciding whether to preserve or transect the TCL. We discovered that a modified Z-lengthening technique with distal transection on the radial side may produce better outcomes compared to a conventional complete TCL incision. However, we also identified that there were no previous studies comparing the modified Z-lengthening with the conventional Simonetta's Z-lengthening technique, highlighting the need for further research in this area.

This study provided a basic understanding of the effect of TCL transection location on CAA and CAC. There are several limitations as follows: one limitation is that the intratunnel pressure was modeled as a constant without considering its interactive effects with TCL transection. The intratunnel pressure could decrease with the increase of CAA after TCL transection. This coupling effect warrants more investigation in the future. Another limitation is that the pseudo-3D FE model focusing on the distal level of the carpal tunnel cannot reflect the volume changes of the carpal tunnel after CTR. Future works can expand the modeling framework developed in this study to be a 3D carpal tunnel model. Finally, the FE model in this study is geometrically specimen-specific, and the future work on population-based modeling can provide more insights into the generalizability of the current results.

## 5. Conclusions

The radial TCL transection on carpal tunnel morphology might be a promising approach for decompression in CTS. Also, TCL transection location on the ulnar half of the TCL might not be a key factor for surgeon's consideration. Our model indicated potential benefit with radial transection location at the distal part the carpal tunnel in a modified Z-lengthening technique.

## Data Availability

The original contributions presented in the study are included in the article, further inquiries can be directed to the corresponding authors.
